# A model of the spatial tumour heterogeneity in colorectal adenocarcinoma tissue

**DOI:** 10.1186/s12859-016-1126-2

**Published:** 2016-06-24

**Authors:** Violeta N. Kovacheva, David Snead, Nasir M. Rajpoot

**Affiliations:** Department of Systems Biology, University of Warwick, Coventry, CV4 7AL UK; Department of HistopathologyUniversity Hospitals Coventry and Warwickshire, Coventry, CV2 2DX UK; Department of Computer Science, University of Warwick, Coventry, CV4 7AL UK; Department of Computer Science and Engineering, Qatar University, Doha, Qatar

**Keywords:** Histology image modelling, Colorectal tissue architecture, Digital pathology

## Abstract

**Background:**

There have been great advancements in the field of digital pathology. The surge in development of analytical methods for such data makes it crucial to develop benchmark synthetic datasets for objectively validating and comparing these methods. In addition, developing a spatial model of the tumour microenvironment can aid our understanding of the underpinning laws of tumour heterogeneity.

**Results:**

We propose a model of the healthy and cancerous colonic crypt microenvironment. Our model is designed to generate synthetic histology image data with parameters that allow control over cancer grade, cellularity, cell overlap ratio, image resolution, and objective level.

**Conclusions:**

To the best of our knowledge, ours is the first model to simulate histology image data at sub-cellular level for healthy and cancerous colon tissue, where the cells have different compartments and are organised to mimic the microenvironment of tissue *in situ* rather than dispersed cells in a cultured environment. Qualitative and quantitative validation has been performed on the model results demonstrating good similarity to the real data. The simulated data could be used to validate techniques such as image restoration, cell and crypt segmentation, and cancer grading.

**Electronic supplementary material:**

The online version of this article (doi:10.1186/s12859-016-1126-2) contains supplementary material, which is available to authorized users.

## Background

Recent popularity of digital slide scanners is generating massive amounts of digital pathology image data [1]. By consequence, the demand for development of robust analytical methods for quantitative morphometric analysis of the histopathology image data is on the rise [2–5]. The uptake of analytical technologies for digital pathology image data depends largely on their ease-of-use and usefulness in terms of accurate quantification. A common approach for validation is to compare the algorithm’s results with expert-labelled data. However, the repeatability and accuracy of expert labelling can be questioned due to human-based error sources [6] and the process is very time-consuming. In order to overcome these difficulties, there is a need for generating virtual (or synthetic) histology imaging data whose spatial characteristics closely match those of the real histology slides and spatial tumour microenvironment therein. In the literature, several frameworks for synthetic fluorescent image data generation have been proposed. One of the earliest works considered the simulation of tissue architecture using graph based methods [7]. More recently, Lockett [8] used a complex set of shapes, such as curved spheres, discs, bananas, satellite discs, and dumbbells. More realistic simulations have also been presented. For example, Lehmussola et al. [9] designed a simulator called SIMCEP, which can simulate large homogeneous 2D cell populations with realistically looking cytoplasm, nuclei and cell organelle. Svoboda et al. [10] generated a model to simulate fully 3D image data of nuclei of cell populations, with realistic distribution [11], and later of healthy colon tissue [12]. However, these models only include cell nuclei. In addition, shape of the nuclei in the colon tissue model of [12] is not very realistic due to the presence of sharp corners generated from the Voronoi diagrams and does not reflect the variety of cell phenotypes found in real tissue. Heterogeneous cell populations expressing different protein markers can be simulated using the SimuCell toolbox [13]. On the other hand, Zhao et al. [14] presented a machine learning method to generate realistic cells with labelled nuclei, membranes and a protein expressed in a cell organelle. However, this approach is restricted to individual cells in culture. The first method for simulating bright-field microscopy was proposed for generating synthetic cytology images of cervical smears [15, 16]. However, tissue microenvironment was not taken into account in that work. Guillaud et al. suggested another in silico approach for understanding tumour architecture by developing a dynamic 3D model of pre-invasive cancer development. However, this method is currently unable to generate realistic microscopy images.

**Fig. 1:**
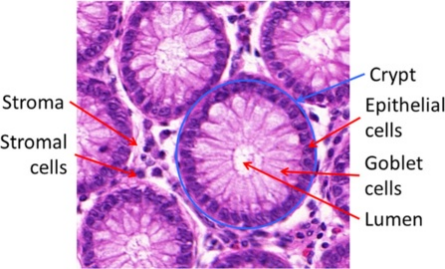
A Hematoxilyn and Eosin (H&E) image depicting the structure of healthy colon tissue
- moderately differentiated
- poorly differentiated
- undifferentiated

Healthy colon tissue microenvironment is composed of a single layer of epithelium forming glandular structures, called crypts (as shown in Fig. 1). The crypts consist mostly of three types of cells: epithelial (absorptive) cells, goblet cells, and stem cells (Fig. 1), and extend down to sit on the *muscularis mucosae*. Goblet cells predominate in the base of the glands, whereas the luminal surface is almost entirely lined by columnar absorptive cells [17]. The tall columnar absorptive cells have oval basal nuclei. In contrast, goblet cell nuclei are small and condensed. There are also stem cells at the base of the crypts, which continuously replace the epithelium. Stroma fills the space between the crypts and contains several types of cells, such as lymphocytes, plasma cells and fibroblasts. As the colorectal adenocarcinoma (CRA) develops from normal tissue, the epithelium exhibits increased dysplasia (pre-malignant change with disordered growth and mutation) and there are fewer mucus-containing goblet cells, reflecting a lack of normal cellular differentiation. Histopathological grading of CRA tumours is performed to provide an indication of their aggressiveness, which is then used for prognosis and/or choice of treatment. The traditional system of grading, also used by the International Union Against Cancer (UICC), is the tumour node metastasis (TNM) [18] classification which distinguishes between four grades of differentiation:

- well differentiated

**Fig. 2:**
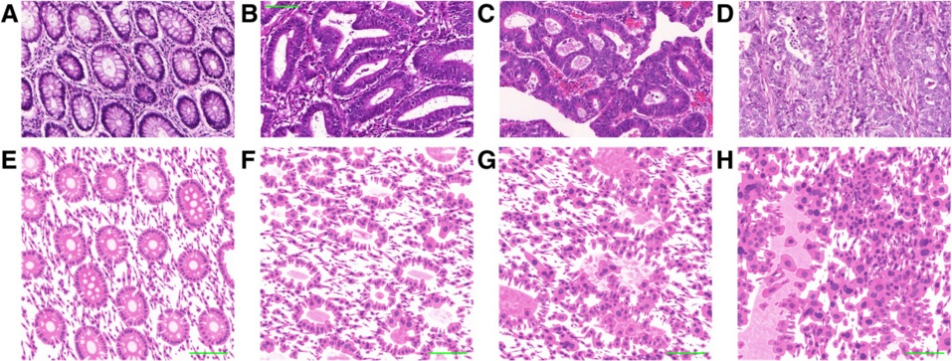
Examples of real (*top row*) and synthetic (*bottom row*) images for different grades: (**a**, **e**) healthy tissue, (**b**, **f**) well, (**c**, **g**) moderately and (**d**, **h**) poorly differentiated cancerous tissue. Images are at 20 × magnification. Size of the scalebars is 100 *μ*m

**Fig. 3:**
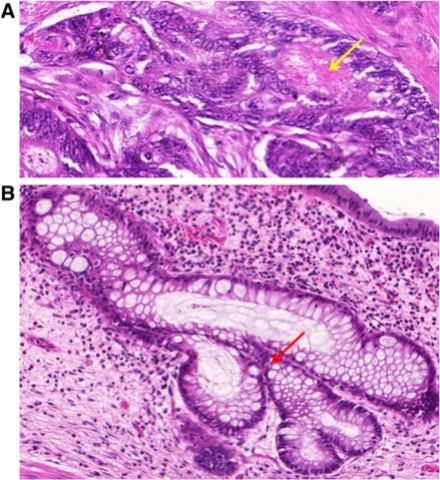
**a** Example of loss of mucin in a moderately differentiated crypt. **b** Example of crypt budding in a benign case

The percentage of tumour showing formation of gland-like structures can be used to define the grade. Well differentiated (grade 1) CRA lesions exhibit glandular structures in >95 % of the tumour; moderately differentiated (grade 2) adenocarcinoma has 50–95 % glands; poorly differentiated (grade 3) adenocarcinoma has 5–50 %; and undifferentiated (grade 4) carcinoma has <5 %. Grades 3 and 4 are often combined, and this convention is followed in this work. There are some additional characteristics that can be used to differentiate between the different grades. Well differentiated tumours have well formed but slightly irregular glands (Fig. 2(b)). Nuclei are basally oriented and exhibit slight atypia, which is characterised by variation in the size of nuclei and visible nucleoli. In moderately differentiated CRAs, there is still a glandular configuration but the glands are irregular and often very crowded (Fig. 2(c)). There can be loss of mucin (Fig. 3(a)) and budding of the crypts (asymmetric crypt division, Fig. 3(b)). One can also observe loss of nuclear polarity and increased nuclei atypia. On the other hand, in poorly differentiated tumours majority of the tumour (excluding the advancing edge) is sheets of cells without gland formation. Some glands may still be observed, but also single cells or clumps of cancerous cells, which are usually bigger than the stromal cells (Fig. 2(d)). Tumour grade is generally considered as a stage-independent prognostic variable, and high grade histology is associated with poor patient survival [19, 20].

In this paper, we propose a model for the spatial microenvironment of healthy and cancerous colon tissue. The Tumour Heterogeneity of Colorectal Tissue (THeCoT) model significantly extends our previous model of the healthy colon tissue [21]. It simulates Haematoxilyn and Eosin (H&E) images of healthy and cancerous colon tissue microenvironment with images for the cytoplasm and cell nuclei. Detailed analysis of real histology images has enabled us to make the model more realistic by extracting parameters for various features such as nuclear and crypt sizes, chromatin and lumen texture, distribution of cell phenotypes, etc. As far as we know, this is the first spatial model for the tumour microenvironment considering different stages of cancer development. The simulated images could be used to objectively compare or train image analysis algorithms. They could be especially useful for pre-training of convolutional neural networks where the high number of parameters needing tuning usually means that an excessive number of hand-marked images is required. While the model may not yet be at a stage to completely replace real hand-marked images, it could be a useful tool to aid validation of image analysis frameworks. The next chapter describes how the model generates the images, starting from the overall architecture and then synthesising each individual cell in turn according to its prescribed phenotype. We then discuss the obtained results and the various methods considered to evaluate the synthetic images.

## Methods

**Fig. 4:**
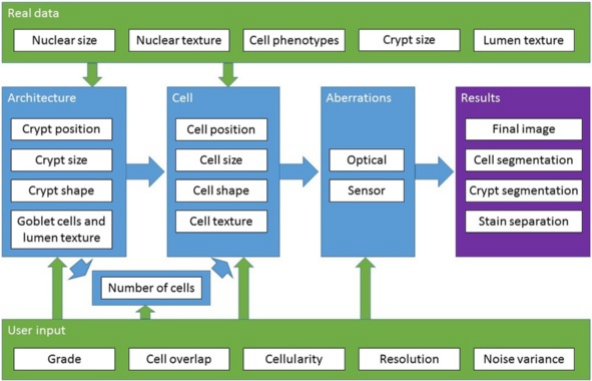
Flowchart of the simulation process. *Blue*, *green* and *purple* boxes contain parts of the model, model inputs and outputs, respectively. The sample grade and crypt sizes from real data input into the architecture generated. The number of cells is determined by the architecture and the user-defined cell overlap and cellularity. Cells are then iteratively generated with input of the cell phenotype distributions and the nuclear sizes and texture found in the real data. Ideal images are then degraded in order to mimic errors in an image acquisition system with parameters of noise variance defined by the user. In addition to the final image, various ground truth data is output

An overview of the model is presented in Fig. 4. The framework is capable of simulating different differentiation grades and has several user-defined parameters to allow control over the tissue appearance in the face of tumour heterogeneity.

### Data acquisition

In order to make the model realistic, H&E slides from colon cancer patients were analysed. The slides were digitally scanned at 40 × magnification by Zeiss MIRAX MIDI Slide Scanner. For cell-level analysis, a total of 42 visual fields at 40 × magnification were considered. These, including a context at 4 × magnification, were graded by three pathologists and the majority vote was taken. The visual fields were categorised as 7 healthy, 4 well-differentiated, 26 moderately differentiated and 5 poorly differentiated samples. Individual nuclei in each image were hand-marked as epithelial or stromal. A total of 5826 nuclei were hand-marked for analysis. In addition, 31 visual fields at 20 × were selected for analysis of the crypt structures. These were split into 9 healthy and 22 cancerous samples. In these, 480 healthy and 396 cancerous crypts were hand-marked. A larger number of cancerous samples were required in order to obtain a similar number of crypts as cancerous crypts tend to be significantly larger. Use of this data is discussed in detail later in the section.

### Tissue structure

In this section we describe how the tissue microenvironment in CRA is modelled. We begin by explaining the overall organisation in terms of the crypts and stroma. We then describe how individual cells are modelled.

#### Crypts

Given an image resolution and magnification level, we assume the appropriate radius, *r*, of the cells to be 6*μm* [22], while a suitable value for the radius of the crypts corresponds to the mean length on the minor axis, *μ*_*b*_, found from the real H&E images and normalised for the magnification and pixel size of the simulation. The generated image depends on the differentiation grade, *S*, of the colorectal adenocarcinoma, which can take the values of 0 to 3, corresponding to healthy tissue (0), well differentiated (1), moderately differentiated (2), and poorly differentiated (3) cancers. The number of crypts and cells to be simulated in the image are determined using their rough sizes. The number of crypts, *N*_*c*_ in an *i*_*h*_×*i*_*w*_ image is determined as follows:

1 $$  N_{c} = f_{c}\lfloor{i_{h}/(2\mu_{b})} \rfloor \lfloor{i_{w}/(2\mu_{b})} \rfloor.  $$

where *f*_*c*_ is the fraction of the sample covered in crypts and is given by

2 $$  f_{c} = \left\{\begin{array}{ll} 1, & \text{if } S = 0, 1 \\ U(0.5, 0.95), & \text{if } S=2 \\ U(0, 0.5), & \text{if } S=3, \end{array}\right.  $$

where *U*(*x*_1_,*x*_2_) is a number uniformly drawn from the range [*x*_1_,*x*_2_]. The value ranges for *f*_*c*_ were determined from pathology guidelines [18] and discussions with pathologists. To create colon tissue structure (Fig. 1), crypts are simulated as elliptical structures. For each crypt, the minor axis *b* is sampled from the Gamma distribution *Γ*(*α*_*b*_,*β*_*b*_), where *α*_*b*_ and *β*_*b*_ are the parameters for the distribution of the minor axis estimated from the real H&E images (see end of “Methods” section) and normalised for the magnification and pixel size of the simulation. To determine the length of the major axis, *a*, we use the ratio between the minor and major axes, *e*=*b*/*a*. Then *a* is given by *b*/(*Γ*(*α*_*e*_,*β*_*e*_)), where *α*_*e*_ and *β*_*e*_ are the parameters for the distribution of *e* (Table 1). The degree of rotation of the major axis, *ϕ*, of the crypts is chosen at random. The crypt outline is then computed as follows,

3 $$ R(\theta) = \frac{a b \sqrt{2}}{\sqrt{(b^{2}-a^{2}) \cos(2 \theta-2 \phi)+a^{2}+b^{2}}} + u,  $$

**Table 1:** Parameters of the model. [ ^∗^ can be variable, depending on real data and magnification level. ^*†*^ typical ranges for 1000 ×1000 pixels image with 40 × magnification, *L*_*max*_=0.6, *ν*_*e*_=*ν*_*s*_=1]

Description	Annotation	Source	Typical values
Image size	*i*_*h*_×*i*_*w*_	User-defined	1000×1000
Magnification		User-defined	40 ×, 20 ×
Size of CCD pixel		User-defined	11 *μ* *m*
Size of scalebar		User-defined	10 *μ* *m*
Cancer grade	*S*	User-defined	{0, 1, 2, 3}
Cellularity of epithelial cells	*ν*_*e*_	User-defined	[0, 1]
Cellularity of stromal cells	*ν*_*s*_	User-defined	[0, 1]
Cell overlap	*L*_*max*_	User-defined	[0, 1]
Variance of point spread function	*G*	User-defined	1 pixel
Variance of the CCD detector noise	*σ*_*G*_	User-defined	0.00025
Stain matrix		User-defined	
Distribution of nuclei major axis length	*μ*_*l*_,*σ*_*l*_	H&E data	^∗^ *μ* *m*
Distribution of nuclei minor axis length	*μ*_*w*_,*σ*_*w*_	H&E data	
Distribution of crypt minor axis length	*μ*_*b*_,*α*_*b*_, *β*_*b*_	H&E data	^∗^ *μ* *m*
Distribution of crypt ratio between axes	*α*_*e*_,*β*_*e*_	H&E data	
Distribution of cell phenotypes		H&E data	
Approximate cell radius	*r*	[22]	6*μ* *m*
Fraction of sample taken by crypts	*f*_*c*_	Eq. 2	[0, 1]
Number of crypts	*N*_*c*_	Eq. 1	
Rotation of crypts	*ϕ*	Random	[0,2*π*]
Cell shape *α*,*β*	Eq. 10	*α*=0.1(*S*+1),*β*=0.05	
Number of epithelial cells	*N*_*e*_	Eq. 8	[110,200]^∗,*†*^
Number of stromal cells	*N*_*s*_	Eq. 7	[150,260]^∗,*†*^
Total number of cells	*N*	Eq. 9	[340,380]^∗,*†*^

where *R*(*θ*) is the polar radius, *θ*∈[0,2*π*] is the polar angle and *u*=(*S*^2^+1)*U*(−0.06,0.1) is a degree of deformation of the crypts, a function of the grade *S*. A small asymmetric range was chosen for *u* to avoid great reductions in the size of the crypts and twisting of the crypt outline.

Then, the crypt centres, **c**=(*x*_*c*_,*y*_*c*_), are selected so that the crypts don’t overlap for healthy or well differentiated samples. For tissues of grades 2 and 3, at most 2 ellipses can overlap to a certain extent. In these cases, one crypt would be modelled by several overlapping deformed ellipses. This generates the “gland within gland” phenomenon and more complex glandular structures often observed in higher grade CRA tissue. In order to speed up the selection of the crypt centre, we only consider a sample of points in a randomly placed grid structure with distance between vertices of 0.6*b*. The epithelial cells are placed at a random location (*x,y*) along or close to the crypt edge location (*x*_0_,*y*_0_) as follows,

4 $$ \begin{array}{rcr} x & = & x_{0} + r S u_{x} \\ y & = & y_{0} + r S u_{y}, \end{array}  $$

where (*x*_0_,*y*_0_) is a randomly selected point on the outline of the crypt, and *u*_*x*_ and *u*_*y*_ are random scaling factors taken from *U*(−0.25,0.08). The scaling factor distribution is taken asymmetric around the crypt outline as to preserve the outline while allowing epithelial cells to be found inside the crypt. It is difficult to extract the exact value of this parameter from real data, so the range was chosen with the aim to maximise visual similarity between real and synthesised images. Hence, in healthy tissue the epithelial cells are attached to the crypt boundary and the structure becomes increasingly distorted for higher differentiation grades. Once the cells are placed, they are rotated so they point towards the crypt centre and, if *S*<2, their nuclei are displaced closer to the edge of the crypt. The stromal cells are placed uniformly in the space outside the crypts. All stromal cells are rotated in a direction given by *ϕ*+*U*(−*π*/6,*π*/6) (Table 1), to reflect the structure of the stromal tissue that can be observed in histology images.

#### Number of cells

The maximum amount of cell overlap is controlled by a parameter *L*_*max*_. The relative amount of overlap, *L*_*ij*_, that is caused on the region of pixels *R*_*i*_ defined by one simulated cell and the region of pixels *R*_*j*_ of another cell is measured by

5 $$ L_{ij} = \frac{|R_{i} \cap R_{j}|}{|R_{i}|}, i \ne j  $$

where |·| is the cardinality of a set. With this definition setting *L*_*max*_=1 doesn’t pose any restrictions on overlap, whereas *L*_*max*_=0 doesn’t allow any overlap. Overlap can be controlled either on the cytoplasm or nuclei. When a cell is placed randomly, if it overlaps with an already placed cell to an extent that is greater than *L*_*max*_, a new set of coordinates is chosen.

In addition to this, in poorly differentiated samples, we place clusters of cancer cells in the stroma. Tumour cells are placed within a cluster in the stromal regions with probability of 50 %. A cluster is a region of size 10*r*×10*r* and cells placed in it have value of maximum overlap equal to *min*(2*L*_*max*_,0.8).

Once the number and size of crypts has been determined and the crypts have been placed, we calculate the number of cells, *N* that will be placed in the image. Firstly, an estimate of the area of a stromal cell, *A* is calculated:

6 $$ A=\pi [(1.7-0.7 L_{max}) r]^{2}.  $$

Here the multiplication factor of *r* accounts for the effect of overlap and doesn’t go below 1 as stromal cells are generally sparse. The area covered by stroma, *A*_*s*_ is found by counting the pixels outside the outlines of the crypts. Then the number of stromal cells is given by

7 $$  N_{s} = \nu_{s} A_{s}/ A,  $$

where *ν*_*s*_∈[0,1] is a user-defined parameter for the cellularity (density) of stromal cells.

Similarly, the number of epithelial cells *N*_*e*_ is determined by

8 $$  N_{e} = \frac{\nu_{e} P }{2(1.25-L_{max}) r}  $$

where *P* is the sum of the perimeters of the crypts in the image, *ν*_*e*_∈[0,1] is a user-defined parameter for the cellularity of epithelial cells, and the factor in the denominator accounts for the effects of overlap. The overlap factor here is smaller than that for stromal cells because epithelial cells are more tightly packed. Then the final number of cells is given by

9 $$  N = N_{s} + N_{e}.  $$

#### Lumen and goblet cells

**Fig. 5:**
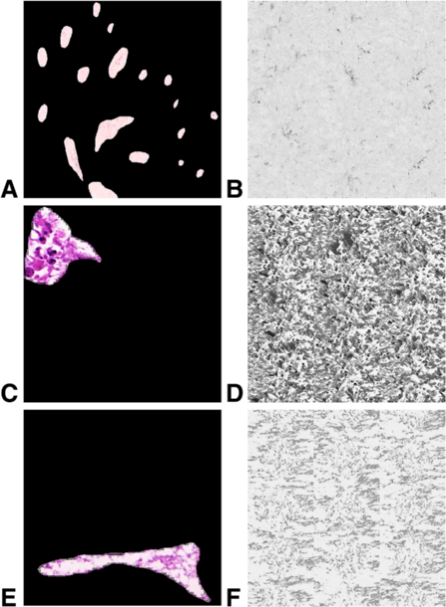
Obtaining lumen texture. Figure (**a**) shows extracted lumen texture from a healthy sample. Figures (**c**) and (**e**) show two of the extracted lumen texture from cancer samples. Figures (**b**), (**d**) and (**f**) show the respective generated texture images

When a sample is being generated, the inside of the crypts is filled with lumen texture. In order to generate the lumen, we employed the non-parametric model [23] which generates texture from a given source image. In this framework, the value of a pixel is determined by finding all patches in the source image that resemble the filled part of the neighbourhood of the pixel in question. One of these patches is selected at random and the value of the centre pixel is assigned to the pixel to be filled. We model the gray-scale texture of hand-marked lumen regions from the real H&E images (see end of “Methods” section) in order to generate a large texture image corresponding to each crypt texture (Fig. 5). Currently, seven textures were generated for cancer crypts and one for normal lumen texture. In the future, this number can be increased to incorporate a wider variety of textures. When a crypt is being synthesised, a random part of a texture image is selected and used as the texture. For healthy samples, the normal lumen texture is used. When a cancer sample is being generated, a texture image is selected at random for each crypt.

**Fig. 6:**
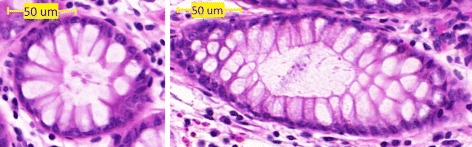
Different goblet cell structures. A roughly circular crypt is shown on the *left* (*κ*=1) and a more elliptical (*κ*=3) on the *right*. Scalebars are 50 *μ* *m*

**Fig. 7:**
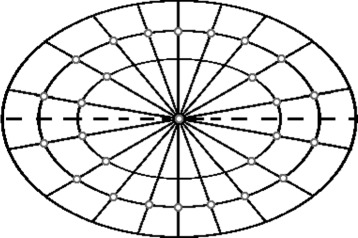
An illustration of the initial locations for the centres of gravity (gray circles) for Voronoi diagram in a crypt with *κ*=2. Dashed line gives the major axis

In healthy samples once the lumen texture is placed, we generate the goblet cells structure. This is done using Voronoi diagrams [24]. The crucial step when generating a Voronoi diagram is to select the centres of gravity for the regions. The observed structure of the goblet cells depends on the angle at which the crypt is sliced through (Fig. 6). Alternatively, we can consider the ratio *e* between the minor and major axes of the crypt as a surrogate indicator of the structure observed. If *e*≈1, (i.e., a round crypt) we get a single ring of goblet cells (Fig. 6(a)). The number of goblet cells in this ring for a particular crypt is given by *γ*=*a*/*r*. However, if *e*<1, we define $\kappa \approx 1/e, \kappa \in \mathbb {N}$, with *κ* rounded to the nearest integer, and we get additional 2*κ*(*κ*−1) goblet cells around each end of the major axis of the crypt. To determine their location, we take even angular increments from the centre of the ellipse and place the points on the outer ring a distance from the crypt boundary equal to the cell radius *r*. The additional points are placed along the 2*κ* angles closest to the major axis a distance 2*i,i*=2,…,*κ* from the boundary (Fig. 7). A centre of gravity for the Voronoi diagram is also added at the centre of the crypt. A small amount of variation is allowed for the location of each point and the Voronoi diagram is generated. To make the boundaries more realistic, they are dilated and the corners at each Voronoi vertex are rounded using dilation. Some texture [25] is added to the boundaries, they are convolved with a Gaussian and added to the final image.

### Single cell

Each of the *N* cells is constructed separately. Before a cell is synthesised, it is assigned to one of the phenotypes found in the real data with probability equal to the probability of the phenotypes in real H&E tissues of the same grade (see end of the “Methods” section). We then generate images for the cell cytoplasm and nucleus as described below.

#### Shape

**Fig. 8:**
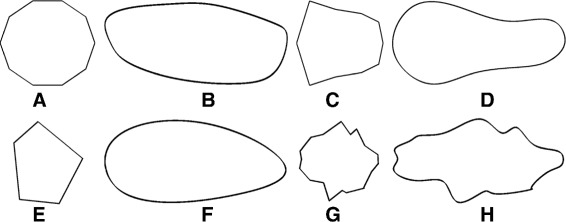
Examples of cell nuclei and cytoplasm shapes. Figures (**a**, **c**) show polygons without any randomness for *k*=10 for the (**a**) stromal and (**c**) epithelial cells. Figures (**b**, **d**) show the corresponding shapes with dislocated vertices after spline interpolation [21]. Figures (**e**) and (**g**) show randomised polygons initialised as circles for *k*=5 and *k*=20, respectively. Figures (**f**) and (**g**) show the corresponding shapes after spline interpolation. Here *α*=0.2,*β*=0.05, *μ*_*l*_=2*μ*_*w*_ and the major axis is shown in the horizontal direction

Two types of shapes are included in our model. First, the cytoplasm for stromal cells and cell nuclei are generated using a parametric model proposed in [9]. In this case, the shapes are initialised as a circle parametrised by (*x*(*θ*),*y*(*θ*)), where *θ*∈[0,2*π*] is the polar angle. The angle *θ* is sampled at *k*(*k*=10) equidistant points to generate a regular polygon (Fig. 8(a)). Then a random polygon is created by randomising the spatial locations of the vertices as follows:

10 $$  \begin{array}{rcl} x_{i}(\theta_{i}) & = & \left[U\left(-\alpha, \alpha\right) + cos\left(\theta_{i} + U\left(-\beta, \beta\right)\right) \right],\\ y_{i}(\theta_{i}) & = & \left[ U\left(-\alpha, \alpha\right) + sin\left(\theta_{i} + U\left(-\beta, \beta\right)\right) \right] \end{array}  $$

for *i*=1,…,*k*, where *α* controls the randomness of the circle radius and *β* controls the randomness of the angle of sampling. The value for *α* is dependent on the cancer grade by *α*=0.1(*S*+1), whereas the value of *β* has been set to 0.05. Taking *k*=10 is a good compromise between taking too few points and not allowing sufficient control over the shape (Fig. 8(d, e)), and taking too many points and obtaining complicated shapes unrealistic for cells in a tissue environment (Fig. 8(f, g)). Then we use the means, *μ*_*l*_ and *μ*_*w*_, and standard deviations, *σ*_*l*_ and *σ*_*w*_, for the nuclei major and minor axes, respectively, obtained from the real H&E data phenotypes and normalised for the magnification and pixel size of the simulation. These are used to obtain the sizes for the modelled nuclei as

11 $$ \begin{aligned} {\mu_{l}^{n}} & = \mathcal{N}(\mu_{l}, \sigma_{l}), \\ {\mu_{w}^{n}} & = \mathcal{N}(\mu_{w}, \sigma_{w}) \end{aligned}  $$

where $\mathcal {N}(\mu, \sigma)$ is the normal distribution with mean *μ* and standard deviation *σ*. Then, the size of the modelled cell cytoplasm is chosen to be

12 $$ \begin{aligned} {\mu_{l}^{c}} & = U(1.5, 2.2){\mu_{l}^{n}}, \\ {\mu_{w}^{c}} & = U(1.5, 2.2){\mu_{w}^{n}} \end{aligned}  $$

The lack of a membrane marker makes it difficult to obtain exact cell size estimates but the interval 1.5–2.2 gives a good approximation of observation from real data (Fig. 2). Normal stromal cells are assigned with equal probability to be either fibroblasts or lymphocytes. For cancer samples, the cells in the stromal regions are assigned to be cancerous with probability 1−−0.2*S*, representing tumour cells infiltrating the stroma. Due to the lack of ground truth, it is difficult to know the exact proportions of tumour cells in the stromal tissue but it is clear that their numbers would increase as the cancer grade increases, the glandular structures break down and the cells obtain more metastatic properties. In order to ensure realistic appearance of the stromal cells, the fibroblast cell sizes are rescaled as

13 $$ \begin{aligned} \hat{\mu}_{w}^{n} & = 0.8{\mu_{w}^{n}}, \\ \hat{\mu}_{l}^{c} & = 1.8{\mu_{l}^{c}}, \\ \hat{\mu}_{w}^{c} & = 0.5{\mu_{w}^{c}} \end{aligned}  $$

$\hat {\mu }_{w}^{n}$ is kept the same, and for lymphocytes as

14 $$ \begin{aligned} \hat{\mu}_{l}^{n} & = 0.8{\mu_{l}^{n}}, \\ \hat{\mu}_{w}^{n} & = 0.8{\mu_{w}^{n}}, \\ \hat{\mu}_{l}^{c} & = 0.7{\mu_{l}^{c}}, \\ \hat{\mu}_{w}^{c} & = 0.7{\mu_{w}^{c}}. \end{aligned}  $$

This generates fibroblast cells with thin nuclei and long and thin cytoplasm, and lymphocytes that are smaller than epithelial cells. The above values were selected to visually resemble the appearance of the real data. Ground truth data on cell functional phenotypes together with a cell membrane marker may enable more accurate estimation of the values. However, such data was not currently available. The cytoplasm of epithelial cells is generated starting from the polygon shown in Fig. 8(c). The set of original coordinates {(*x*_*i*_,*y*_*i*_),*i*=1,…,*k*} is then scaled as follows,

15 $$ \begin{aligned} \hat{x}_{i}(\theta_{i}) & = x_{i}(\theta_{i})\mu_{l}^{n/c},\\ \hat{y}_{i}(\theta_{i}) & = y_{i}(\theta_{i})\mu_{w}^{n/c}. \end{aligned}  $$

where *μ*^*n*/*c*^ refers to both *μ*^*n*^ and *μ*^*c*^. Finally, the vertices are interpolated using cubic splines (Fig. 8(b) and (d)).

#### Texture

**Fig. 9:**
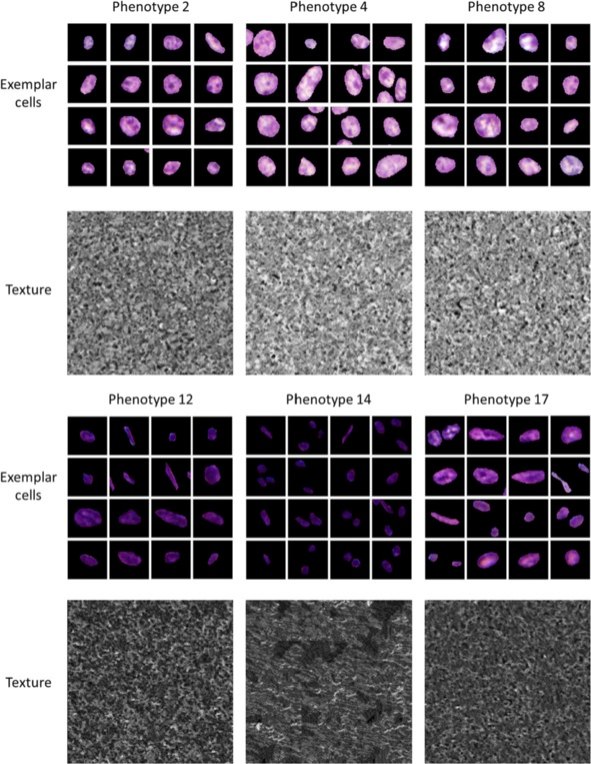
Selection of cells belonging to different phenotypes with corresponding texture images below. The phenotypes shown are numbers 2, 4, 8, 12, 14, and 17 from Fig. 11. One can easily see that the first row of phenotypes contains mostly tumour and epithelial cells, whereas the second one consists mostly of stromal cells

Texture for the cytoplasm is generated using a well-known procedural model [25] for texture synthesis. The nuclear chromatin texture is an important factor when grading cancers and has been shown to relate to cancer stage [26]. Hence, a more sophisticated method was adopted for synthesising it. In particular, we used the non-parametric model presented by Efros and Leung [23]. The model is applied to the grey-scale texture of all the nuclei found to belong to the real phenotypes in order to generate a large texture image (Fig. 9 (b, d)). When a nucleus of a particular phenotype is being synthesised, a random part of the corresponding texture image is selected and used as the nuclear texture. The sampling is done with replacement, and hence, although unlikely, two nuclei could have the same texture. Although this texture synthesis method produces more realistic results, it is very computationally expensive and so its use has been limited within the model. Texture images and sample of cells belonging to the corresponding phenotype for several of the phenotypes found in the real data are shown in Fig. 9. The same method is also used to generate the lumen texture shown in Fig. 5.

**Fig. 10:**
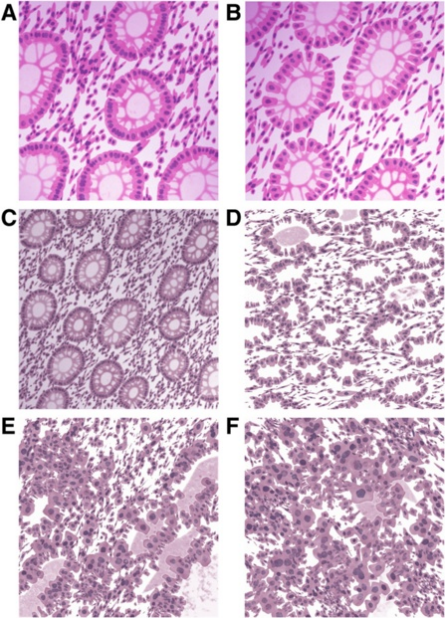
Examples of synthesised images demonstrating the effects of different parameter values. Figures (**a**)–(**c**) show examples of simulated images of healthy colon tissue. The images are 1000×1000 pixels, at magnification (**a**, **b**) 40 × and (**c**) 20 ×. In figure (**b**) the overlap *L*_*max*_=0.2 and the cellularity *ν*_*s*_=*ν*_*e*_=1. All other figures have *L*_*max*_=0.6 and the cellularity *ν*_*s*_=*ν*_*e*_=1. Figures (**d**)–(**f**) show various differentiation grades. The images are 1000×1000 pixels, at magnification 20 ×. The figures show (**d**) well differentiated, (**e**) moderately differentiated, and (**f**) poorly differentiated cancers. Figures (**a**) and (**b**) were generated using the stain vector proposed by [27], whereas the rest of the Figures were generated using the stain matrix in Eq. 17

**Fig. 11:**
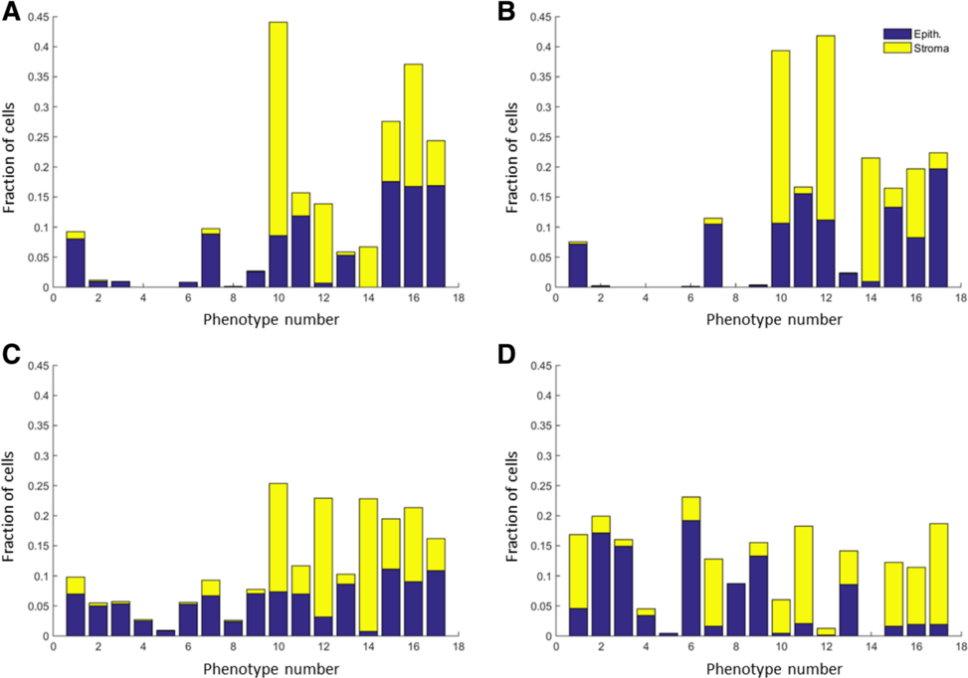
Frequency of each type of cell belonging to a phenotype. Clustering of the nuclear texture features has been performed on the real hand-marked nuclei. **a** shows frequencies for healthy epithelial and stromal cells. **b–d** show frequencies for well, moderately and poorly differentiated, respectively

### Measurement error

The final step of the simulation degrades the ideal images constructed in the previous sections. This resembles the degradation caused by the real measurement system. Firstly, convolution with a 2D Gaussian, *G*, is used to simulate the leaking of photons between neighbouring pixels. We also add zero mean Gaussian noise, *N*_*G*_ with variance *σ*_*G*_ to approximate the CCD detector noise (Table 1). Hence, the simulated image degraded by the acquisition system, $\hat {I}$, obtained from an ideal image *I* is given by:

16 $$ \hat{I} = I*G + N_{G},   $$

where ∗ denotes the convolution operator.

### Histology Simulation

The generated cytoplasm and nuclei channels are converted into H&E stains (Fig. 10) using a user-defined colour deconvolotion matrix. In the results for this paper we used the colour deconvolotion matrix suggested by Ruifrok and Johnston [27] and a matrix obtained from an image using the stain separation method proposed by Trahearn et al. [28] as follows:

17 $$  M= \left[\begin{array}{lll} 0.6402 & 0.6479 & 0.4128 \\ 0.3906 & 0.7662 & 0.5102 \end{array}\right].  $$

By simulating immunohistochemistry stains, the usability of the model is expanded to verification of a wide range of methods for analysis of H&E images. As one can choose the stain vector used to generate the images, the model can be utilised to validate stain normalisation methods, such as Khan et al. [29]. In addition, H&E images can be easily assessed by pathologists who routinely deal with histology slides.

Lastly, the user can choose to add a scalebar of desired length to the generated image. Given the magnification and objective level the model calculates the length in pixels and inserts the bar at the bottom right corner of the image (Fig. 2).

### Learning from the real data

**Fig. 12:**
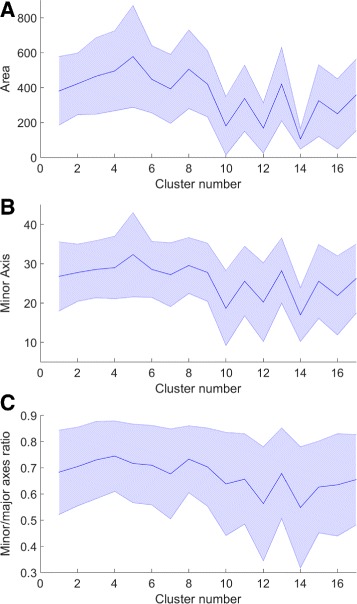
Size feature profiles for nuclear texture phenotypes found in the real data showing (**a**) nuclear area, (**b**) minor axis length and (**c**) minor/major axes ratio. The graphs show mean ± standard deviation. Sizes are in pixels for 40 × images

We perform detailed analysis of the real H&E data described at the start of the section in order to extract some of the parameter values used within the model. This enables us to make the model more realistic. As whole-cell segmentation is difficult to obtain from H&E slides, we concentrate on studying the nuclear regions. This approach is supported by findings that the nucleus can hold the key to understanding cell function [26, 30]. In order to extract nuclear information visual fields at 40× magnification were analysed. Size and 13 Haralick texture features were extracted for each nucleus. Affinity Propagation [31] was used to phenotype the nuclei according to the textural features, in order to group together cells with similar texture without requiring to input the number of clusters. For each of the 17 phenotypes found in this way, mean and standard deviation of the length of the major axis and the ratio between the minor and major axes were obtained (Table 1). In addition, we calculated the frequency with which nuclei belonging to each phenotype were found to be epithelial or stromal, for incorporation of the phenotype frequency into our model as described above. These frequencies are shown in Fig. 11. Some of these phenotypes were found to contain mostly cancerous epithelial nuclei (Fig. 9 top rows), whereas others consisted of predominantly stromal nuclei (Fig. 9 bottom rows). The average profiles for size and texture features are shown in Fig. 12 and the Additional file 1, respectively. In addition, we obtained hand-marked images for crypt texture. One image was used to obtain healthy lumen texture (Fig. 5(a)). Seven crypts from different cancer samples were also marked and texture was extracted. Figure 5(c) and (e) show two of these.

**Fig. 13:**
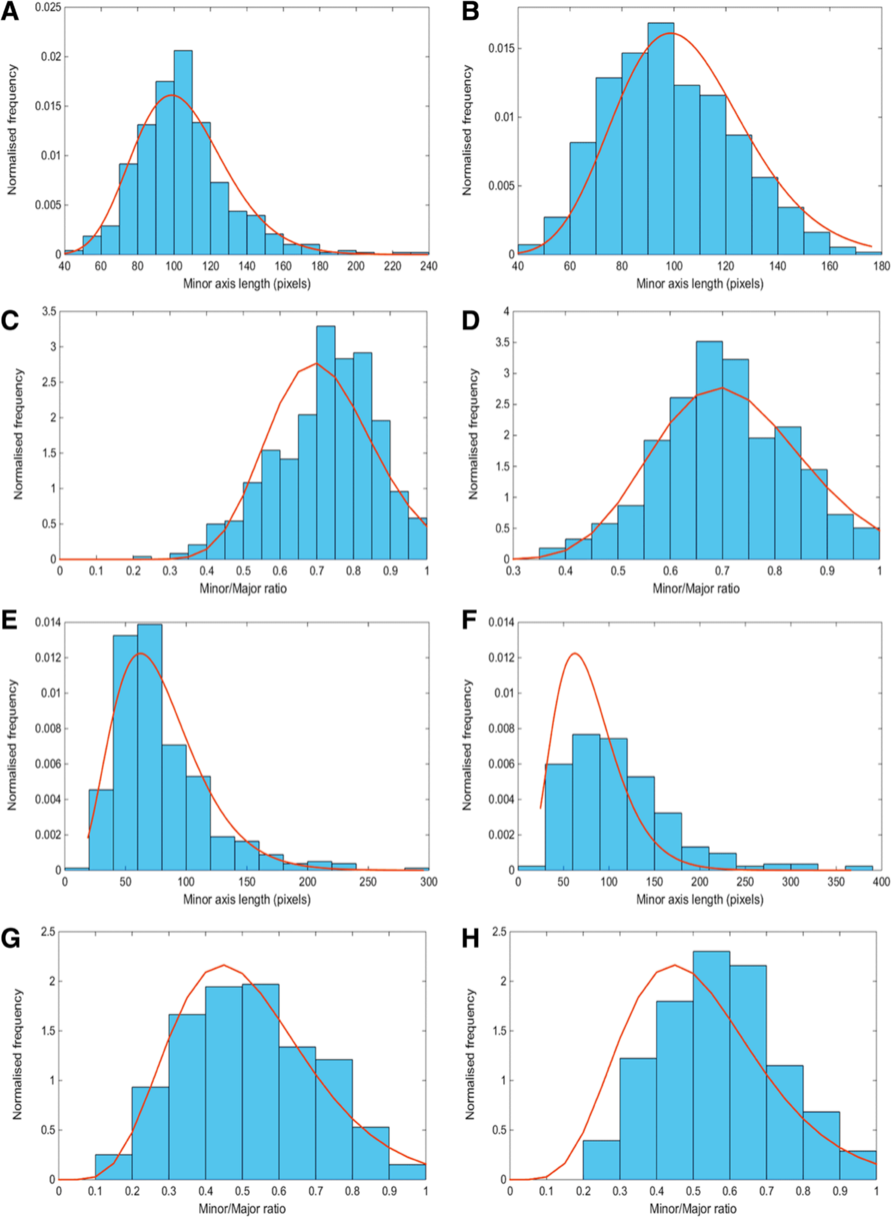
Distribution of crypt size parameters extracted from the real (left column) and synthetic (*right column*) data. Figures (**a**) and (**b**) show the minor axis length for healthy crypts. Figures (**c**) and (**d**) show the ratio between the minor and major axes for healthy crypts. Figures (**e**) and (**f**) show the minor axis length for cancerous crypts. Figures (**g**) and (h) show the ratio between the minor and major axes for cancerous crypts. Frequencies are normalised so that sum of areas of bars equals 1

In addition to this, visual fields at 20× magnification were selected for the analysis of crypt shapes and sizes. We calculated the distributions of the minor axis and the ratio between minor and major axes for each group. These were modelled as Gamma functions and the parameters were incorporated into the model. The fit of the Gamma distributions is shown in Fig. 13.

## Results and discussion

THeCoT models the tumour heterogeneity in colorectal tissue. Examples of the resulting images are shown in Figs. 2 and 10. There are several user defined parameters which allow control over the appearance of the imaged tissue. Figures 10(a) and (b) illustrate how changing the parameters for overlap and cellularity affects the resulting images. Figures 10(c) – (f) show how the tissue structure changes as the differentiation grade is increased. When the user specifies the cancer grade, there is a number of parameters integrated as part of the model that also change. These include the size, shape and appearance of the crypts, whether or not the nuclei are basally orientated, and the frequency of cell phenotypes (Table 1). It is worth noting that in the model we assume that Eosin is highly specific to marking the cytoplasm. While in reality this is not necessarily the case, the lack of a membrane marker in the ground truth data makes it difficult to separate and model the non-specific binding. It is easy for the user to experiment with different parameters as the model takes around 108 s to simulate a 40 × image and around 345 s for a 20 × image. Both of these times are average over 10 runs to generate healthy images with overlap of 0.6 and cellularities of 1. The times were recorded when simulations were run on a PC equipped with Intel Core i5-4310U 2 Ghz processor and 16 GB RAM. The code is executed serially, hence, if a large number of images is required, multiple simulations could be run in parallel.

### Cell segmentation

**Fig. 14:**
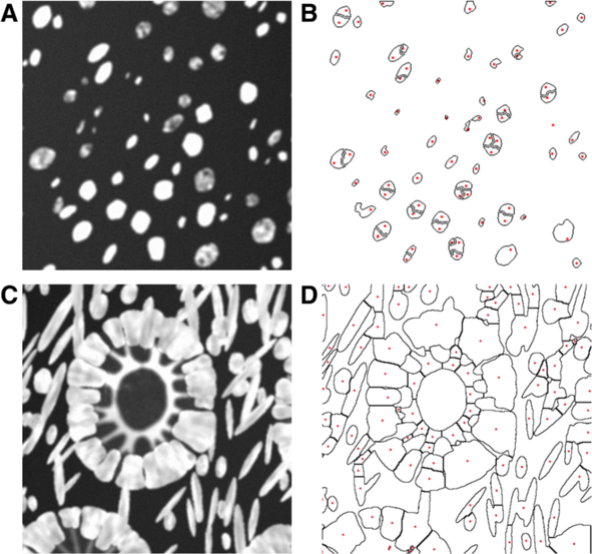
Examples of segmentation results using ImageJ. Figures (**a**) and (**b**) show original data for a cancerous image and results from nuclear identification using the ITCN plugin followed by watershed segmentation to obtain nuclear boundaries. Red dots mark centres of detected regions. Figures (**c**) and (**d**) show original data for a healthy image and results from segmentation using thresholding and watershed segmentation. Figures (**b**) and (**d**) show the borders of the segmented regions with a red dot identifying the centres of the proposed segmented cells

Manipulating parameters for cell overlap and cellularity could be very important when testing cell segmentation algorithms, for instance. Depending on the purpose for image synthesis, one may require to have fewer, easily separable cells, or more crowded and overlapping cells. The results from cell counting experiments, similar to the ones in [9, 21], using ImageJ [32] and CellProfiler (CP) [33] are shown in Table 2. Cell counting was done on a total of 20 simulated samples, 10 healthy and 10 moderately differentiated cancerous images at 40 × magnification, and cellularities *ν*_*s*_=*ν*_*e*_=1. It was performed both on the non-overlapping nuclei regions and on the cytoplasmic regions where overlap of 0.4 was allowed. In CP segmentation was performed by first using an Otsu thresholding with an adaptive threshold. When performing nuclei segmentation minimising the weighted variance gave the best results. However, for segmenting the overlapping cytoplasms, minimising the entropy gave better results and these are reported in Table 2. Objects outside the diameter range [8, 50] pixels for nuclei and [8, 100] pixels for cytoplasm were considered mis-segmented and hence were discarded. In ImageJ, two different approaches of segmentation were adopted. Firstly, cells were counted using the ITCN (Image-based tool for counting nuclei) Plugin for ImageJ developed by Thomas Kuo and Jiyun Byun at the Center for Bio-image Informatics at UC Santa Barbara [34]. Its algorithm assumes nuclei to be blob-like structures with roughly convex local intensity distributions whose iso-level contour is approximately ellipsoidal; nuclei are fitted by an inverted Laplacian of Gaussian filter [34]. Images were inverted before using ITCN. Cell detection was performed by detecting dark peaks with the following parameters: cell width = 22, minimum distance = 4, threshold = 1. This method was unable to segment the cytoplasmic images due to their more complex shapes. Hence, a second method for segmentation was tested where the images were first thresholded manually and then watershed was used to attempt to segment regions further. We can see that CellProfiler performed significantly better on the healthy than the cancerous images due to the more consistent nuclei sizes. Similar behaviour was observed for ImageJ using both segmentation algorithms, with cell counting results closer to the ground truth for the healthy images. However, we can see from Fig. 14 that, in fact ITCN tended to over-segment larger nuclei while missing smaller ones (Fig. 14(b)). On the other hand, watershed under-segmented cells but picked up regions of the goblet cells cytoplasmic architecture (Fig. 14(d)). This is confirmed further by the large under-segmentation of the cancerous images. It is important to note that above algorithms may perform better with further tuning of their parameters. This study only aimed to demonstrate how such algorithms could be compared based on performance on the synthetic data generated by THeCoT.

**Table 2:** Cell counting results for CellProfiler (CP) and ImageJ(IJ) with ITCN (Image-based tool for counting nuclei) Plugin and with watershed segmentation. Counting based on non-overlapping nuclei or cytoplasm regions with *L*_*max*_=0.4. Mean ± standard deviation are shown normalized by the ground truth. A value over 1 shows over-segmentation, whereas a value under 1 demonstrates under-segmentation

CellProfiler (CP) and					
Image type	CP nuclei	CP cytoplasm	IJ nuclei ITCN	IJ nuclei	IJ cytoplasm
All	1.007 ± 0.014	0.919 ± 0.149	0.952 ± 0.036	1.094 ± 0.041	0.945 ± 0.283
Healthy	1.014 ± 0.011	1.046 ± 0.084	0.976 ± 0.022	1.062 ± 0.023	1.139 ± 0.291
Cancer	1.001 ± 0.015	0.792 ± 0.071	0.929 ± 0.031	1.125 ± 0.029	0.751 ± 0.021

### Evaluation by pathologists

To assess how realistic the appearance of the synthetic histology images generated by the model is, we asked three pathologists to grade them. They were presented with images for the four tissue grades, at magnifications of 40 × and 20 × and with cell overlap of 0.2 and 0.6 (total of 16 images). They consistently rated the number of crypts, epithelial and stromal cells as realistic, suggesting that this is a suitable range for the overlap parameter. Grades for the appearance of the tissue are shown in Table 3. The average grade given was 4.28 out of 5. The pathologists on the whole graded the stromal cells as being less realistic. This is because while one could tell they are stromal cells, one couldn’t determine what type of stromal cells they were.

**Table 3:** Average evaluation of the appearance of synthetic images by 3 pathologists. Healthy (H), well differentiated (WD), moderately differentiated (MD), and poorly differentiated (PD) images were evaluated at magnifications 20 × and 40 ×. (1 = Not realistic at all, 5 = Very realistic, ’-’ means feature is not relevant)

	H	H	WD	WD	MD	MD	PD	PD
	40 ×	20 ×	40 ×	20 ×	40 ×	20 ×	40 ×	20 ×
Architecture	5	5	5	4	4	4	5	5
Crypt shape	5	5	5	5	5	5	4.5	4.5
Lumen	5	5	5	5	5	5	-	-
Goblet cells	4	4	-	-	-	-	-	-
Epithelial cells	4	4	4	4	4	4	4	4
Stromal cells	3	3	3	3	3	3	3	4

### Crypt architecture

The most distinguishing characteristic of the colon microenvironment is the crypt structure. An earlier version of the model was validated by comparing the means and standard deviation of morphological features of the synthesised healthy crypts with those calculated from the hand-marked histology images [21]. Here we have expanded this by looking at the overall distributions. We found excellent agreement between the distribution of the minor axis length and the ratio between the minor and major axes and the Gamma distributions estimated from the real data. The results are shown in Fig. 13. Our earlier work also demonstrate how the model could be used to compare different cell segmentation algorithms. In order to evaluate the overall appearance of the crypt structure, we utilised a crypt segmentation method proposed by Sirinukunwattana et al. [3]. We generated a database of 15 images for each grade (60 in total). The H&E images were generated using a stain vector of a real image used to train the crypt segmentation method. The stain vector was determined using the method proposed by [28]. The results for the Dice coefficient on both pixel-level and object-level are shown in Table 4. To calculate the evaluation indices, we let the *g* be a set of pixels marked as ground truth and *o* a set of pixels segmented as glandular structures. Then the Dice index is given by

18 $$ Dice(g,o) = \frac{2|g \cap o|}{|g| + |o|}.  $$

**Table 4:** Pixel-level and object-level dice coefficient for crypt segmentation of synthetic images of various grades at 20 × magnification. Crypts were segmented using a thresholded probability map method [3]. Results are shown when the method was trained and tested on the synthetic and on real data. The reported figures are the average ± standard deviation

Training data	Test	Grade	Dice-Pixel	Dice-Object
Synthetic	Synthetic	Healthy	0.96 ± 0.003	0.91 ± 0.03
		Well	0.94 ± 0.005	0.90 ± 0.03
		Moderately	0.91 ± 0.02	0.90 ± 0.03
		Poorly	0.65 ± 0.15	0.65 ± 0.13
Real	Synthetic	Healthy	0.87 ± 0.01	0.85 ± 0.02
		Well	0.89 ± 0.01	0.84 ± 0.03
		Moderately	0.88 ± 0.11	0.52 ± 0.11
		Poorly	0.59 ± 0.16	0.36 ± 0.11
Synthetic	Real	Benign	0.69 ± 0.11	0.53 ± 0.13
		Moderately	0.58 ± 0.16	0.43 ± 0.13
		Poorly	0.60 ± 0.17	0.44 ± 0.17

For the object-level segmentation accuracy, let *o*_*i*_ denote the set of pixels of the *i*th segmented object in *o* and *g*_*i*_ denote the set of pixels of ground truth objects in *g* that intersect *o*_*i*_. Further, let $\hat {g}_{i}$ denote the set of pixels of the *i*th ground truth object in *g* and $\hat {o}_{i}$ denote the set of pixels of segmented objects in *o* that intersect $\hat {g}_{i}$. Then the object-level Dice index is defined as

19 $$ Dice_{obj}(g,o) = \frac{1}{2}\left[ \sum_{i=1}^{n_{o}} \omega_{i} Dice(g_{i}, o_{i}) + \sum_{i=1}^{n_{g}} \hat{\omega}_{i} Dice(\hat{g}_{i}, \hat{o}_{i}) \right],  $$

where $\omega _{i} = |o_{i}|/\sum _{j=1}^{n_{o}}|o_{j}|$, $\hat {\omega }_{i} = |\hat {g}_{i}|/\sum _{j=1}^{n_{o}}|\hat {g}_{j}|$, and *n*_*o*_ and *n*_*g*_ are the total number of segmented and ground truth objects, respectively. Hence, the object-level Dice index is always at most the pixel-level Dice index.

Most of the results are comparable with results for real data [3]. The method performs worse for high grade cancerous samples when trained and tested on different datasets. This is likely to be due to the fact that the segmentation framework relies heavily on the texture within and outside the cancerous crypts. The model currently does not include the extra-cellular matrix which generates the texture between the stromal cells. In addition, the model may need a wider variety of textures available for inside the cancer crypts.

### Chromatin texture

**Fig. 15:**
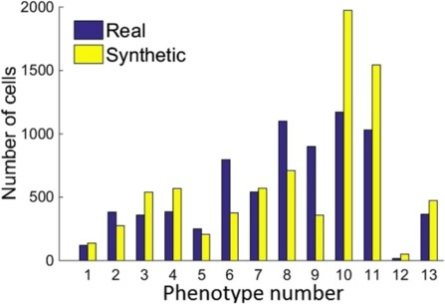
Number of cells of each phenotype of real and synthetic nuclei when clustering is performed based on their texture

A further set of 20 images (10 healthy and 10 moderately differentiated) were simulated at 40 × with an average of 360 cells per image. In order to check that the synthesis of nuclear texture has produced satisfying results, we analysed the nuclei of the 20 synthetic images described above and the hand-marked nuclei from real H&E images. The Haralick features of all the nuclei were calculated and these were phenotyped using Affinity Propagation. As can be seen in Fig. 15, the synthetic data produces a similar distribution of nuclear phenotypes as compared to that of the real data. This demonstrates the suitability of the framework adopted for chromatin texture synthesis. In addition, we can see that the distribution of the phenotypes of the real and synthetic nuclear textures are nearly equal.

## Conclusions

We presented a model for simulating healthy and cancerous colonic tissue architecture at the microscopic scale. Modelling the tumour microenvironment for CRA allows us to better understand some of the underlying laws such as the distributions of cell phenotypes and changes in the tissue architecture. The proposed model has several parameters, which allow control over the tissue appearance. Detailed analysis of hand-marked H&E images has enabled us to make the model realistic by learning parameters to generate realistic cell phenotypes, chromatin and lumen texture, nuclei morphology, and crypt architecture. To the best of our knowledge, ours is the first model to simulate histology image data of cancerous tissue, where the cells are organized to mimic the microenvironment of tissue *in situ* as opposed to dispersed cells in a cultured environment. Majority of features of the histology images produced by the model have been rated as being very realistic by pathologists. We have also shown an example of how a crypt segmentation method can be used on the synthetic data. In addition, we have demonstrated that phenotyping of the cells on the basis of their textural characteristics showed consistency in the results for both real and synthetic nuclei. While the synthesised data may not yet be realistic enough to fully replace real data in the process of validating image analysis techniques, it could be a useful tool and may reduce the need for a large number of real images needed. The model could aid the development of techniques such as image restoration, cell and crypt segmentation, stain normalisation, and cancer grading. It could also be of great use for pre-training convolutional neural networks. In future, we plan to improve the model by separately modelling the types of cells found in colorectal tissue. We would also include a model for the extracellular matrix and other phenomena observed in cancerous tissue, such as necrosis. In addition, the model could be extended to include expression of multiple proteins of interest, in order to simulate multiplex fluorescence or immunohistometry images. This could further aid the study of tumour heterogeneity.

## Abbreviations

CRA, colorectal adenocarcinoma; H&E, haematoxylin and eosin; THeCoT, tumour heterogeneity of colorectal tissue

## Additional file

Additional file 1 File textFeat.xsl. Texture feature profiles for phenotypes found in the real data. Phenotypes are obtained based on the Haralick texture features shown in the table. For each feature the mean is shown in the first column and the standard deviation in the second column. (XLSX 17 kb)
